# *Latilactobacillus sakei* LB-P12 Ameliorates Osteoarthritis by Reducing Cartilage Degradation and Inflammation via Regulation of NF-κB/HIF-2α Pathway

**DOI:** 10.4014/jmb.2504.04013

**Published:** 2025-05-02

**Authors:** Mikyung Song, Won Jun Kim, Jaeseok Shim, Kyoungsub Song

**Affiliations:** R&D Center, LISCure Biosciences Inc., Seongnam 13488, Republic of Korea

**Keywords:** Osteoarthritis, *Latilactobacillus sakei* LB-P12, NF-κB, HIF-2α

## Abstract

Osteoarthritis (OA) is a degenerative joint disease characterized by cartilage degradation, inflammation, and pain. Recent studies highlight the gut-joint axis, suggesting that gut microbiota influences joint health by modulating systemic inflammation and immune responses. This study investigated the effects of *Latilactobacillus sakei* LB-P12 on cartilage degradation and joint inflammation in a monosodium iodoacetate induced rat model of OA. OA severity was assessed through histological analysis, weight-bearing and micro-computed tomography (Micro-CT). Serum Interleukin 6 (IL-6) and prostaglandin E2 (PGE_2_) levels, along with interleukin-1β (*Il1b*) and matrix metalloproteinase 13 (*Mmp13*) expression in knee tissue, were measured. Then, the effect of *L. sakei* LB-P12 on inflammatory responses in interleukin-1β pretreated chondrocytes has also been investigated. The *L. sakei* LB-P12 improved weight-bearing distribution and reduced cartilage damage based on histological scores. Micro-CT showed increased bone volume fraction and bone mineral density. Treatment reduced serum IL-6 and PGE_2_ levels and suppressed *Il1b* and *Mmp13* expression in knee tissues. *In vitro*, *L. sakei* LB-P12 inhibited lipopolysaccharide induced pro-inflammatory cytokines and nitric oxide production in macrophages. It also downregulated the expression of *Epas1*, which encodes hypoxia-inducible factor-2α (HIF-2α), and *Mmp13* in IL-1β stimulated chondrocytes. *L. sakei* LB-P12 shows potential as a dietary supplement for alleviating OA-related pain, cartilage degradation, and inflammation by suppressing the nuclear factor-κB (NF-κB)/ HIF-2α pathway.

## Introduction

Osteoarthritis (OA) is a degenerative joint disease and the most common form of arthritis characterized by the degradation of the articular cartilage, joint inflammation, which lead to chronic pain and progressive movement disability [1]. Although OA affects more than 500 million individuals globally, there is no cure to treat or prevent the disease progression and the only available treatments are pain relievers, anti-inflammatory medications, or replacement arthroplasty [2, 3]. Currently, non-steroidal anti-inflammatory drugs (NSAIDs), corticosteroids, and acetaminophen are widely used for reducing pain and inflammation, these medications are mainly focused on symptomatic relief rather than restoring or preventing the cartilage damages in OA patients. Nutraceuticals such as glucosamine, methylsulfonylmethane (MSM), and chondroitin sulfate have also been used as dietary supplements supportive of joint health, yet their efficacy remains debated [4-7].

The pathogenesis of OA is a complicated process that involves mechanical, inflammatory, and metabolic factors, which eventually lead to structural destruction and failure of the synovial joint [8]. Cartilage degradation is the most well-known feature of OA, and chondrocyte is the major cellular component that regulates homeostasis through the synthesis and degradation of the cartilage proteins. Notably, the progression of cartilage degradation in OA is mediated by activation of catabolic tissue proteinase such as matrix metalloproteinase (MMPs), collagenase, and aggrecanase, which are released from chondrocytes and immune cells [9]. Synovial inflammation has also been observed in over half of OA patients at both early and late stages of disease [10]. Macrophages are the most abundant immune cell types in the OA synovium, and they secrete pro-inflammatory cytokines and inflammatory mediator respond to environmental factors including lipopolysaccharide (LPS), interferon gamma (IFN-g) and tumor necrosis factor-a (TNF-a). These inflammatory factors induce a chondrocyte catabolic process which lead to extracellular matrix (ECM) degradation through the upregulation of MMPs [11]. Over the decades, several cellular signaling pathways have been proposed that involved in the onset and progression of OA, including the canonical *Wnt*, Notch, JAK/STAT, nuclear factor-κB (NF-κB), and HIF pathways [12]. All these signaling cascades play a critical role in cartilage development and homeostasis, and synovial inflammation.

Gut microbiome has been associated with a wide range of diseases, including inflammatory bowel diseases, obesity, metabolic disorders, cancer and autoimmune diseases [13]. It is now well accepted that the gut microbiome contributes to chronic local and systemic inflammation by regulating host immune cells or intestinal barrier functions [14]. In recent year, several studies have shown that the worsening of OA can be prevented by modulation of gut microbiota based on the concepts of the gut-joint axis [15]. The study showed that treatment of antibiotic could alleviate the progression of OA in mice [16], and the serum and synovial fluid lipopolysaccharide levels were closely related to OA severity and inflammation [17]. Interestingly, growing evidences showed that gram negative bacterial DNA presents in synovial fluid and tissues of patients with OA [18, 19]. Probiotics, widely regarded as live microorganisms, have been reported to have the capacity to positively influence gut health and regulate immune responses when administered in adequate amounts [20]. Several studies have demonstrated that specific probiotic strains, including *Lacticaseibacillus rhamnosus*, *L. casei*, and *Lactobacillus acidophilus*, could ameliorates the progression of OA by reducing pain, inflammatory responses, and articular cartilage degradation [21-24].

In this study, we examined the effect of *Latilactobacillus sakei* LB-P12 on the progression of OA in monosodium iodoacetate (MIA)-induced rat model of OA. Both the *in vitro* and *in vivo* data suggest that *L. sakei* LB-P12 effectively suppressed the pathogenesis of OA by decreasing the expression of pro-inflammatory cytokines and MMPs in macrophage and chondrocytes. The results of this study provides the evidences of the capacity of *L. sakei* LB-P12 to alleviate OA and their potential underlying mechanisms.

## Materials and Methods

### Materials

Lipopolysaccharide (LPS, *Escherichia coli* O55:B5, L5418) and human recombinant Interleukin-1β (IL038), Monoiodoacetate (MIA) were purchased from Sigma-Aldrich (USA). Interleukin-6 (IL-6) and Prostaglandin E2 (PGE_2_) ELISA kits were purchased from R&D Systems (USA). MSM was kindly provided by Natural way (Republic of Korea). TRIzol reagent was obtained from Invitrogen (USA). cDNA synthesis kit (K2563) and SYBR Green PCR master mix (4364346) were obtained from Thermo Fisher Scientific (USA). The nitric oxide detection kit (21023) was purchased from from iNtRoN Biotechnology Inc., (Republic of Korea).

### Preparation of *Latilactobacillus sakei* LB-P12

*L. sakei* LB-P12 was isolated from kimchi and the stock of *L. sakei* LB-P12 was deposited with accession number KCTC 13818BP at Korean Collection for type cultures (KCTC, Republic of Korea). *L. sakei* LB-P12 was kept as frozen stock in MRS Broth (de Man, Rogosa and Sharpe, BD Difco, USA) containing 30% (v/v) glycerol at –80°C, until use. To thaw the frozen stock, *L. sakei* LB-P12 was incubated in MRS broth at 30°C for 24 h, and then harvested by centrifugation (685 ×*g* for 20 min). The cell pellets were washed, and resuspended in sterile phosphate-buffered saline (PBS) for *in vitro* assays and animal studies.

### Animals and Induction of OA Procedures

Fifty male Sprague-Dawley rats were obtained from ORIENT BIO Inc., (Republic of Korea). The rats were acclimated for 7 days and housed under controlled conditions at a temperature of 22 ± 2°C, humidity of 55 ± 15%, a 12 h light /12 h dark cycle, and fresh-air ventilation (10–15 times/h). The rats were housed in solid-bottom cages with free access to food and water. The animals were randomized and assigned to treatment groups prior to the start of the study. All experimental procedures complied with the National Institute of Health Guide for the Care and Use of Laboratory Animals and the Korean National Animal Welfare Law. The experimental animal facility and study protocols were approved by the Institutional Animal Care and Use Committee of DT&CRO (DTE230027). After anesthetization with isoflurane, rats were injected with 50 μl containing 3 mg of MIA (Sigma) using a 26.5-G needle inserted through the patellar ligament into the intra-articular space of the right knee. Control rats were injected with an equivalent volume of saline. The vehicle-treated animals were given an equivalent volume of saline. After the injection of MIA, the rats with MIA-induced osteoarthritis received the Low (1 × 10^8^ CFU/head) and High (1 × 10^9^ CFU/head) doses of *L. sakei* LB-P12 for 28 days. MSM was used as positive control. After sacrificing the rats, blood was collected and centrifuged (3,000 ×*g*, 15 min) for plasma collection. IL-6 and PGE_2_ in plasma were determined using commercial ELISA kits (R&D Systems) according to the manufacturer’s protocols.

### Assessment of Weight Bearing

Before MIA injection, a weight bearing index was measured using a static weight bearing test device (SWB-TOUCH-R, Bioseb, France). OA was induced in 8-week-old male Sprague Dawley rats (*n* = 10/each group) by MIA injection. The animals were placed in an inclined acrylic chamber, and the hind legs were placed on separate pressure sensors, respectively. The force applied to each hind leg was measured three times for 10 seconds, after then calculate the average value. Weight bearing were measured on Day 3, 7, 14, 21 and 28. The individual data point was the average of three measurements. The percentage of weight divided onto the handled (ipsilateral) hind limb was calculated utilizing the following equation: (weight on right leg/weight on right leg and left leg) × 100.

### Micro-CT Analysis

Analysis of periarticular bone was performed using a vivaCT 80 (Scanco Medical, Switzerland). The scanner setting was X-ray source voltage 70 kVp and 114 μA, 8 W. The bone parameter was measured by BMD (bone mineral density, g/cm^3^), bone volume fraction (Bone volume, BV/Tissue volume, TV ; BV/TV%). Following acquisition, the images were reconstructed using the Skyscan NRecon programme, and analyzed using the Skyscan CTAn software.

### Histological Analysis

Histological changes were assessed to determine the effects of *L. sakei* LB-P12 treatment in the knee joint of rats. The animals were perfused via the ascending aorta with 10% neutral buffered formalin (pH 7.4). The knee joints, including the patella and joint capsule, were resected and maintained in the same fixative for an additional 48 h at 4°C. The fixed knee joint specimens were decalcified with 5% formic acid for 6 days at 4°C, and then embedded in paraffin. Standardized 4 mm serial sections were obtained at the medial and lateral midcondylar level in the sagittal plane and stained with Hematoxylin-Eosin (KPNT, Republic of Korea) and Safranin O (Scytek, USA). After staining, the degree of articular cartilage lesions was scored by Mankin scoring [25, 26]. The score ranges from 0 to 14; higher scores indicate more severe joint and cartilage destruction.

### Real-Time Polymerase Chain Reaction (RT-PCR)

Total RNA was extracted from knee cartilages of rats, Raw 264.7 cell and SW1353 chondrocytes according to the protocol described for the TRIzol reagent (Invitrogen). cDNA synthesis was performed using a cDNA synthesis kit (K2563, Thermo Fisher Scientific), after then qRT-PCR was conducted using QuantStudio Real-Time PCR (Thermo Fisher Scientific) with the SYBR Green PCR master mix (4364346, Thermo Fisher Scientific). The PCR cycling conditions were as follows : pre-denaturation one cycle at 95°C for 1 min denaturation at 95°C for 10 sec, annealing at 60°C (depending on the primers used) for 40 cycles, and elongation and fluorescence data collection at 60°C for 30 sec [27]. Glyceraldehyde 3-phosphate dehydrogenase (GAPDH) was used as an internal control. The primer sequences are enlisted in Supplementary Table S1. The gene expression was quantified in comparison to those of the negative control group.

### Cell Culture

The murine macrophage cell line Raw 264.7 cells was obtained from the Korean Cell Line Bank (KCLB, Republic of Korea) and SW1353 chondrocytes (HTB-94) were obtained from the ATCC (American Type Culture Collection, VA, USA). The Raw264.7 and SW1353 chondrocytes were cultured in high glucose Dulbecco’s Eagle’s Medium (DMEM; Gibco, USA) supplemented with 10% fetal bovine serum (Thermo Fisher Scientific), 1%penicillin (100 U/ml) and streptomycin (100 μg/ml). Raw264.7 cells were seeded at a density of 1.5 × 10^5^ cells per well, and SW1353 cells at 6.0 × 10^4^ cells per well in 24-well plates. Then, both cells were cultured at 37°C in a 5%CO_2_ atmosphere (3111, Thermo Fisher Scientific Forma); the cultures were replaced every 2 days. The Raw264.7 cells were treated with *L. sakei* LB-P12 of multiplicity of infection (MOI) 0.5 (7.5 × 10^5^ CFU/ml) and 1 (1.5 × 10^5^ CFU/ml) in the presence or absence of LPS (100 ng/ml) for 24 h. The SW1353 cells were also treated with *L. sakei* LB-P12 MOI 0.5 (3.0 × 10^4^ CFU/ml) and 1 (6.0 × 10^4^ CFU/ml) with or without IL-1β (10 ng/ml). The production of nitric oxide (NO) was measured in the supernatant of Raw264.7 cells using NO assay kit (21023, iNtRON Biotechnology Inc.) as following manufacture’s instruction. The produced NO was measured using a microplate reader (Multiskan SkyHigh, Thermo Fisher Scientific) at wavelength of 450 nm.

### Statistical Analysis

Statistical analysis was performed using GraphPad Software (Prism 10, USA). All data are presented as the mean ± S.E and SEM. Biochemical parameters and gene expression data and results from animal histological study were analyzed by a Student’s *t*-test. In all analyses, *p* < 0.05 was taken to indicate statistical significance.

## Results

### *L. sakei* LB-P12 Relieves OA-Associated Pain

**Fig. 1:**
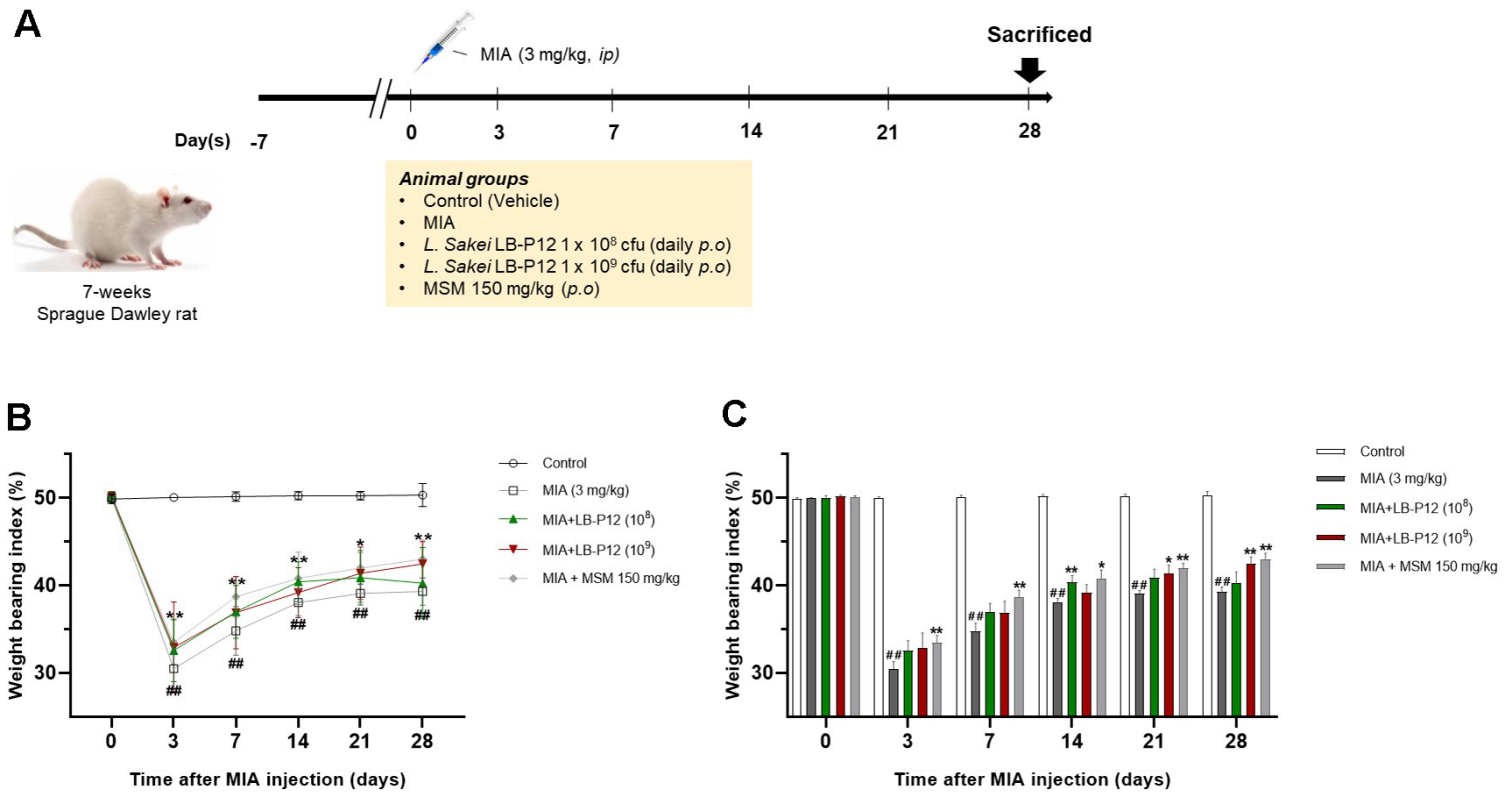
*L. sakei* LB-P12 relieves OA-associated pain. (**A**) Schematic timeline of the experimental procedure. Sevenweek- old rats were randomly divided into five groups. After one week acclimated, MIA was injected into the intra-articular space of right knee as described in *Material & Methods*. The rats received daily oral gavage of two different doses of *L. sakei* LBP12 (1 × 10^8^ CFU/head or 1 × 10^9^ CFU/head) or 150 mg/kg of MSM for 28 days. (**B-C**) Weight bearing was measured on Day 0, 3, 7, 14, 21 and 28. Data are presented as the mean ± SEM. #*p* < 0.05 and ## *p* < 0.01, Control vs MIA group and *p < 0.05 and **p < 0.01, MIA vs MSM and LB-P12 treated group.

To investigate the effects of *L. sakei* LB-P12 on osteoarthritis, intra-articular MIA injection model was used in this study. The MIA model of OA in rat is a well-established and widely used chemical model, characterized by a robust and rapid pain phenotype. Rats were divided into a total 5 groups (*n* = 10), and *L. sakei* LB-P12 or MSM (used as a positive control) was administered for 28 days via daily oral gavage (Fig. 1A). Pain-relieving effects were evaluated by measuring weight-bearing activity on days 3, 7, 14, 21, and 28 after MIA injection. The weight-bearing index (WBI) were rapidly reduced by MIA injection and remained low compared to the normal group during experimental periods, indicating the model was well developed. As shown in Fig. 1B and 1C, high-dose of LB-P12 resulted in significantly increase of WBI compared to MIA group on day 21 (*p* < 0.05) and day 28 (*p* <0.01). Low-dose of LB-P12 showed trends in increase WBI compared to MIA group, but this was not statistically significant. The MSM treated group also showed significant increase in WBI compared to MIA group, but there were no significant differences compared to the high-dose LB-P12 group. All treated group showed no significant difference of body weights between groups (Fig. S1). These results suggest that orally administered *L. sakei* LB-P12 effectively alleviate OA-related pain in rats, as indicated by significant WBI improvement independent of body weight changes.

### *L. sakei* LB-P12 Reduces the Cartilage Destruction associated with OA

**Fig. 2:**
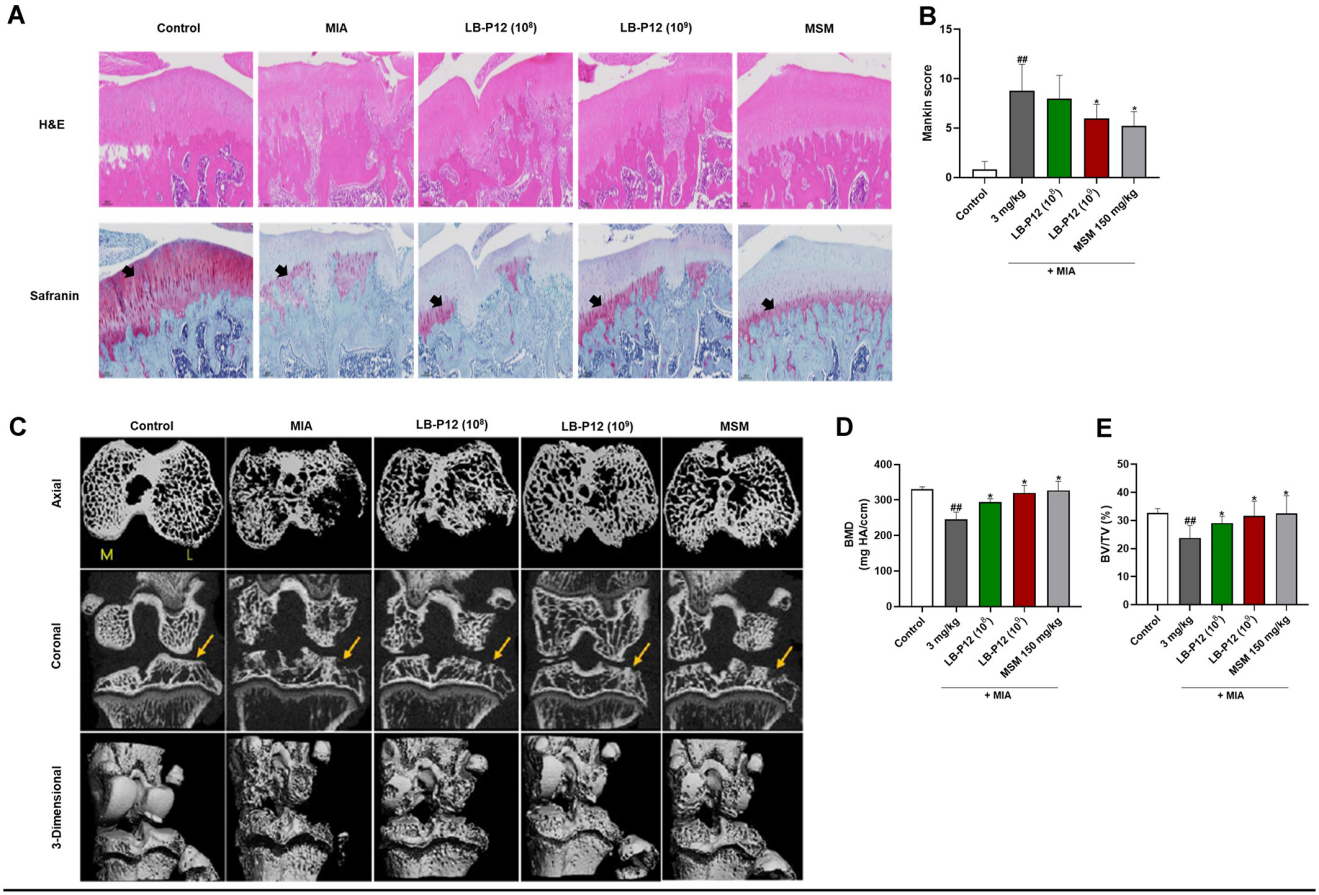
*L. sakei* LB-P12 reduces bone and cartilage degradation in MIA-induced OA rats. (**A**) Representative images of H&E‐ and Safranin O‐stained knee joints from vehicle‐treated MIA‐induced and LB-P12‐treated MIA‐induced OA rats. Note the normal cartilage structure of the joint in the rats that appeared as red pigmentation (black arrows). The images for each group were obtained under × 80 magnification. (**B**) Quantitative analyses of Mankin scores. (**C**) Visualization of osteophytes using micro-CT coronal and 3D images. (**D-E**) Quantitative analyses of bone mineral density (BMD, mg HA/ccm) and bone volume fraction (BV/TV, %). Data are presented as the mean ± SEM. ## *p* < 0.01, Control vs MIA group and * *p* < 0.05, MIA vs MIA-treated LB-P12 (10^9^ CFU/head) by *t*-test by using Prism GraphPad 10 (**USA**).

To assess whether *L. sakei* LB-P12 has beneficial effects by improving cartilage tissue damages, histological analysis was performed using Hematoxylin and Eosin (H&E) and Safranin O-staining. The control group did not show any remarkable lesions in the articular cartilage. In the MIA-treated group, the red-stained normal cartilage was destroyed by MIA and the proteoglycan layer disappeared. In contrast, the proteoglycan layer was clearly seen in the groups treated with high dose LB-P12 and MSM. The representative images are shown in Fig. 2A. The cartilage degradation was further evaluated by Mankin score system which assesses four parameters, cartilage structure, cellularity, Safranin O staining, and tidemark integrity [25, 26]. The Mankin score of normal control group was 0.8 ± 0.37, showing the normal cartilage shape. At 28 days after MIA injection, the Mankin score was markedly increased in the MIA treated group (8.8 ± 1.20) when compared to control group (*p* < 0.0001). In contrast the high dose LB-P12 (1 × 10^9^ CFU/head) treated group showed significant lower Mankin score (6.0 ± 0.63) compared to MIA only group (*p* < 0.05) in Fig. 2B. These results indicate that *L. sakei* LB-P12 may protect against cartilage degradation. Next, we performed Micro-CT (vivaCT 80, Scanco Medical Bruettisellen, Switzerland) analysis to assess micro-architectural alterations in MIA-induced OA and to evaluate the potential protective effects of *L. sakei* LB-P12. We observed the femur and tibia regions around the cartilage. Coronal and Axial micro-CT images revealed marked subchondral bone loss in the MIA group compared with controls, whereas minimal loss was evident in the *L. sakei* LB-P12 high-dose-treated rats (Fig. 2C). Analyses of bone mineral density (BMD) and bone volume (BV) revealed that the MIA group had significantly decreased BMD and BV compared to the control group. The BMD and BV/TV (%) values were the lowest in the MIA-induced group at 246.30 ± 20.03 mg HA/ccm and 23.82 ± 1.96%, respectively, showing significant differences compared to the control group. In contrast, the high-dose LB-P12 group showed significant improvement compared to the MIA-induced group (BMD; 320.08 ± 21.02 mg HA/ccm and BV/TV; 31.67 ± 2.33%, respectively). (Fig. 2D and 2E). The MSM used as a control substance showed BMD and BV/TV (%) comparable to the high-dose LB-P12 group. These results suggest that *L. sakei* LB-P12 treatment significantly improvement cartilage destruction induced by MIA.

**Fig. 3:**
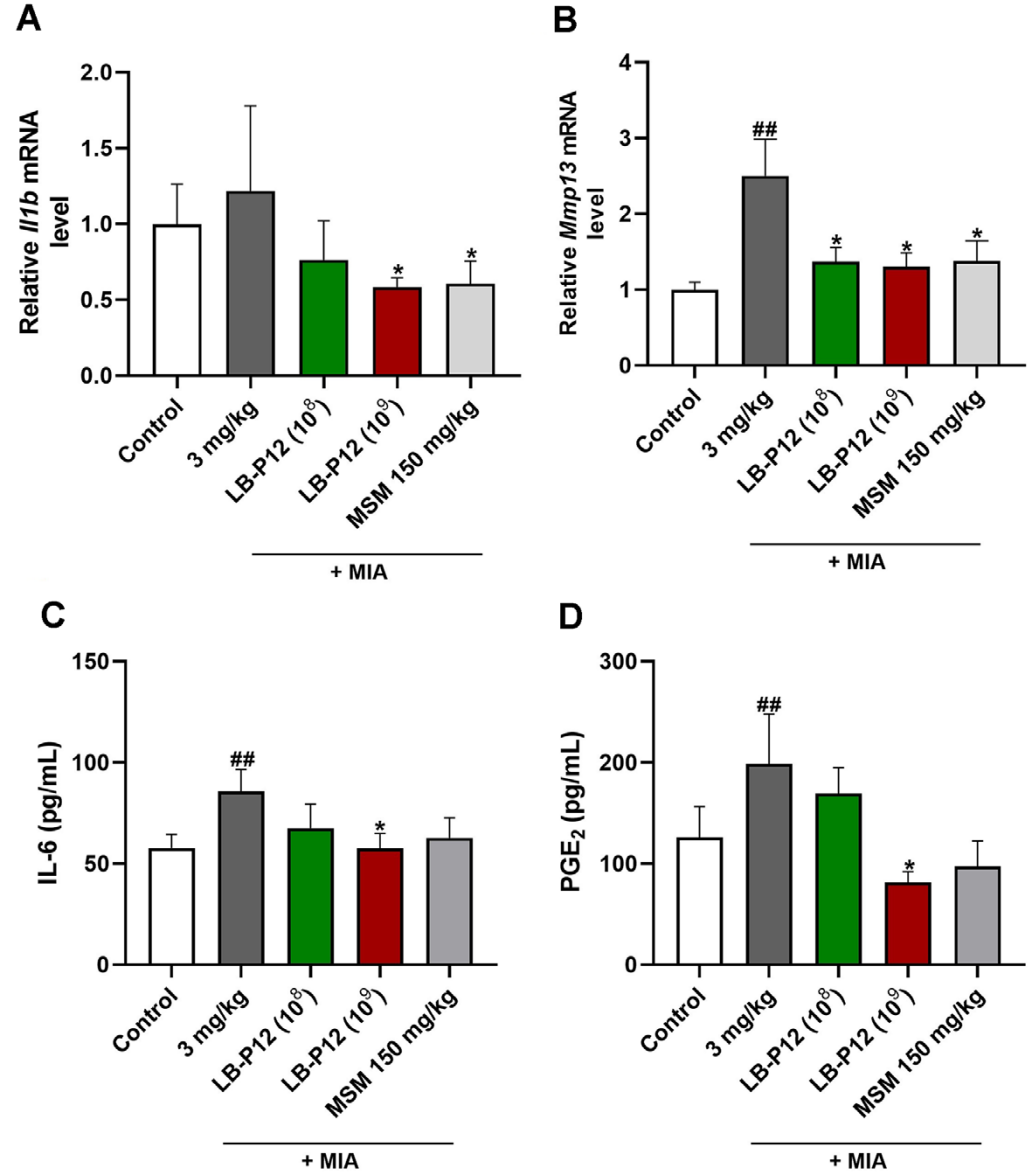
The *L. sakei* LB-P12 reduces OA-mediated inflammation in MIA-induced OA rats. (**A, B**) RNA was extracted from the cartilage tissue of OA rats after sacrifice at Day 28, and the mRNA levels of *Mmp13* and *Il1b* were determined by qRT-PCR analysis. (**C-D**) The serum levels of IL-6 and PGE_2_ in OA rats were measured using enzyme-linked immunosorbent assay (**ELISA**) kits. Data are presented as the mean ± SEM. Statistical significance is indicated as follows: #*p* < 0.05 and ##*p* < 0.01 vs. Control vs MIA group and *p < 0.05 MIA vs MIA-treated with MSM and LB-P12 (10^8^ CFU/head) and LBP12( 10^9^ CFU/head) by *t*-test by using Prism GraphPad 10 (USA).

*L. sakei* LB-P12 Inhibits the Levels of Inflammatory Mediator and Catabolic Factor in MIA-Induced OA Rats The inflammatory response is a critical factor associated with OA pathogenesis, elevated proinflammatory mediator were frequently observed in OA patients. In addition, it has been known that these proinflammatory cytokines induces catabolic factors such as matrix metalloproteinase in chondrocytes in the joint [28]. To evaluate whether LB-P12 administration affects in local-articular and systemic inflammation, we examined the levels of proinflammatory cytokines and catabolic factor, MMP-13 in joint tissues and plasma in MIA-induced OA Rats. Our qPCR analysis revealed that the mRNA levels of inflammation genes (*Il1b*) and extracellular matrix degradation genes (*Mmp13*) were increased in MIA group compared to vehicle group. The administration of LB-P12 (1×10^9^ CFU/head) and MSM downregulated the levels of these genes compared to MIA group (Fig. 3A and 3B). In addition, the serum levels of IL-6 and PGE_2_ were also significantly decreased by treatment of LB-P12 (Fig. 3C and 3D). These results suggest that *L. sakei* LB-P12 may protect the cartilage degradation by reducing local joint and systemic inflammation.

### *L. sakei* LB-P12 Regulates the Levels of Inflammatory Mediators and Catabolic Factor through NF-κB/HIF-2α in Macrophages and Chondrocytes

**Fig. 4:**
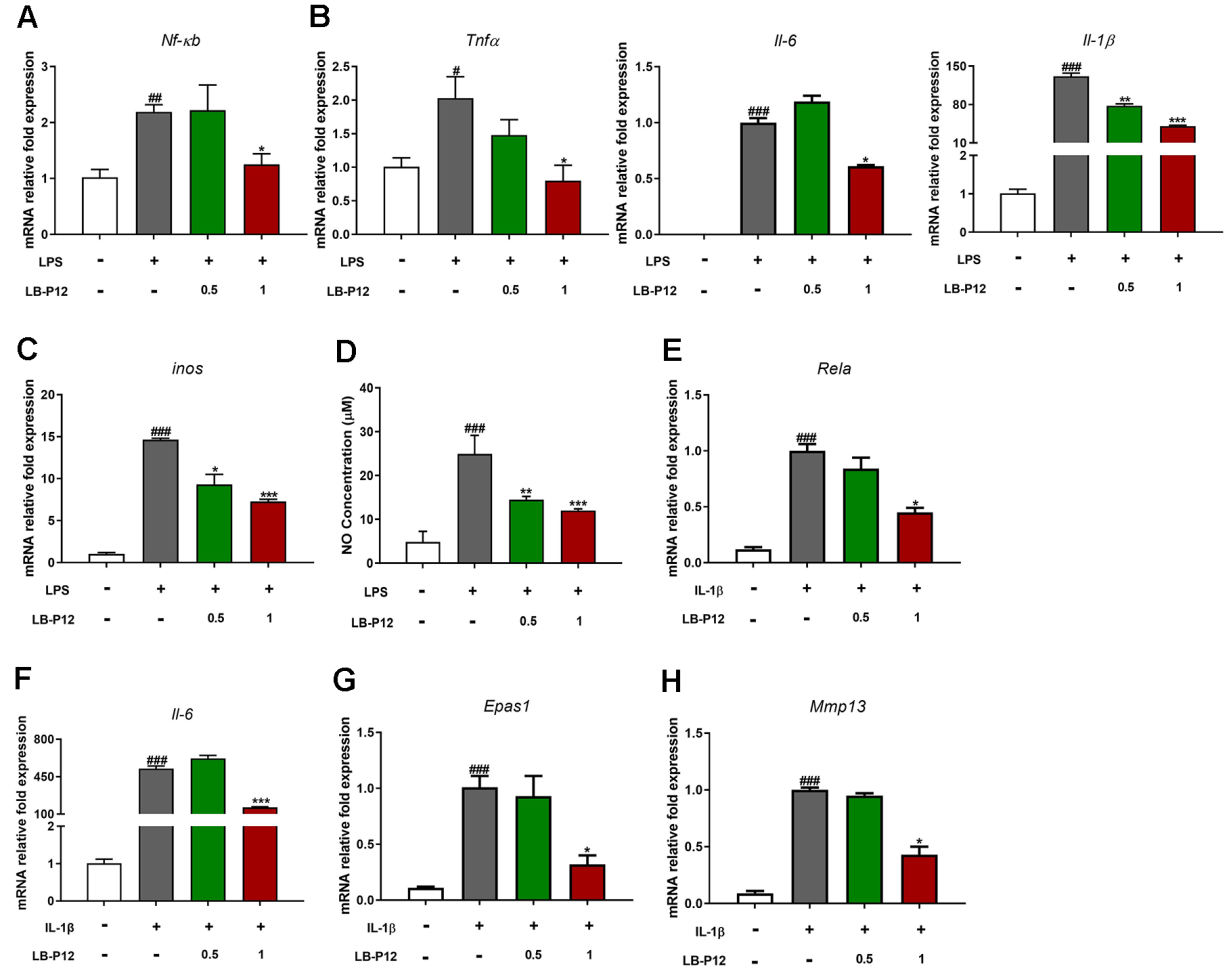
Regulation of proinflammatory cytokine, matrix metalloproteinase and their transcription regulator NF-κB/HIF-2α in macrophages and chondrocytes. Raw 264.7 cells and SW1353 cells were treated with *L. sakei* LB-P12 (MOI 0.5 and 1) in the presence of LPS (100 ng/ml) and IL-1β (10 ng/ml) for 24 h, respectively. mRNA expression levels of the indicated genes were measured RT-qPCR. GAPDH was used as endogenous control. (**A**) Treatment with *L. sakei* LB-P12 significantly suppressed LPS-induced Nf-kb expression in Raw264.7 cells. (**B**) Expression levels of proinflammatory cytokine genes (Tnf-α, Il6, and *Il1b*) were markedly reduced following *L. sakei* LB-P12 treatment in Raw264.7 cells. (**C**) *L. sakei* LB-P12 also inhibited LPS-induced inos gene expression in Raw264.7 cells. (**D**) The production of NO was measured in the supernatant of Raw 264.7 cells using NO detection kit as described as *Material & Methods*. (**E-H**) The expression levels of *Rela* (NF-κB p65 subunit), *Il6*, *Epas1* (encoding HIF-2α), and *Mmp13* were measured in IL-1β-treated SW1353 chondrocytes with or without LB-P12 treatment. All data are presented as mean ± SD from three independent experiments. Statistical analysis was performed using *t*-test with GraphPad Prism 10 software: *p < 0.05, **p < 0.01 and ***p < 0.001 compared to negative control group; #*p* < 0.05, ##*p* < 0.01, ###*p* < 0.001 compared to control.

Macrophage-mediated inflammation and chondrocytes degeneration play a critical roles in OA. To investigate how *L. sakei* LB-P12 regulates inflammation and cartilage destruction, we examined the effects of *L. sakei* LB-P12 on LPS-induced macrophages and IL-1β-induced chondrocytes. In the Raw 264.7 murine macrophage cell, the mRNA levels of proinflammation cytokines (*Il6*, *Tnf*, *Il1b*, and *Nos2*) and their transcriptional regulator *Nfkb* were significantly inhibited when it compared to LPS group (Fig. 4A-4C). In addition, nitric oxide (NO) production was also significantly decreased compared to LPS group in a dose-dependent manner (Fig. 4D). In similar with Raw264.7 cells, treatment of *L. sakei* LB-P12 reduced the levels of *Nfkb* and Il6 expression that induced by IL-1β in SW1353 human chondrocytes.

**Fig. 5:**
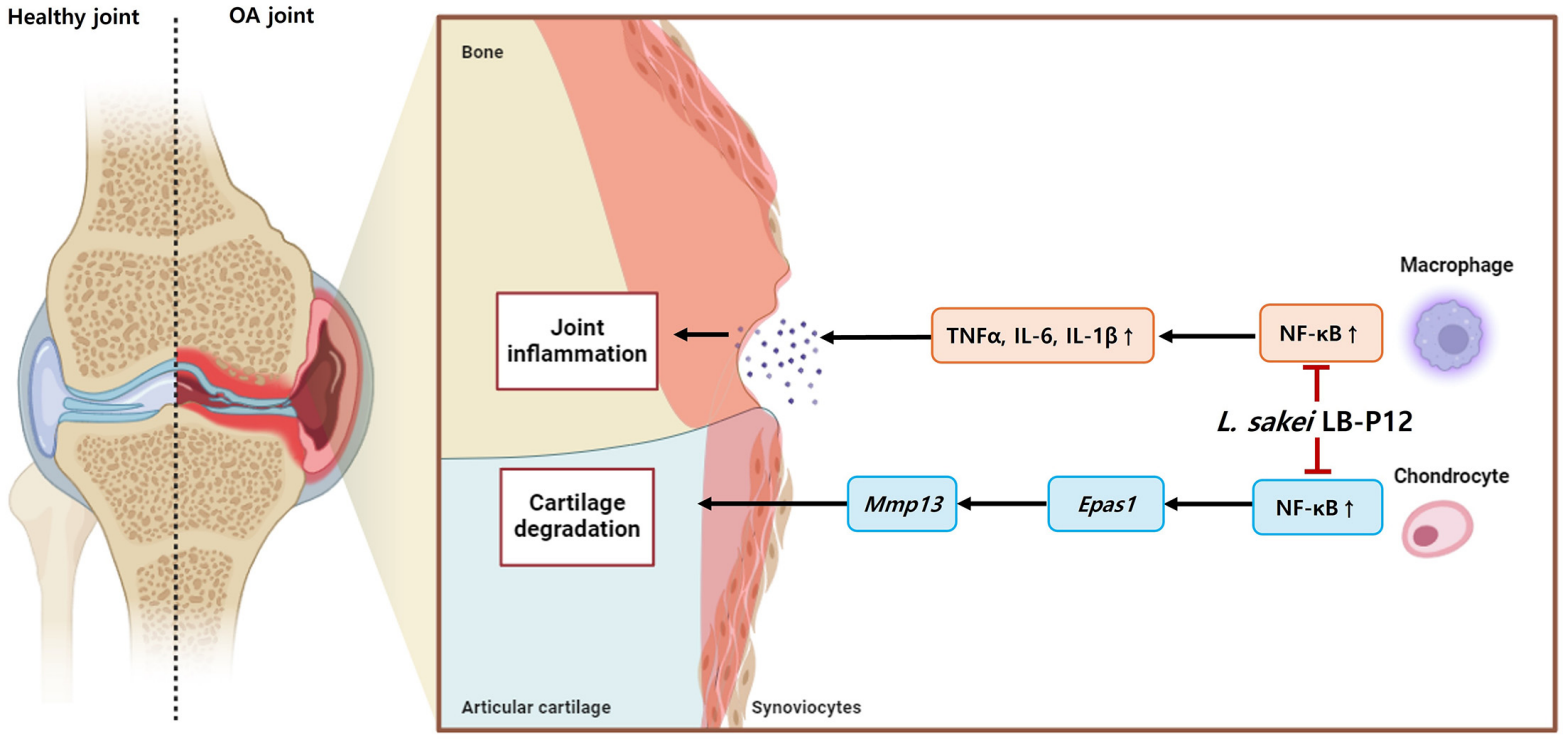
Proposed mechanisms of action for *L. sakei* LB-P12 in OA. A schematic illustration showing the proposed mechanisms by which *L. sakei* LB-P12 ameliorates OA pathogenesis. In macrophage, *L. sakei* LB-P12 may inhibit the production of proinflammatory cytokines by suppressing NF-κB signaling pathway. In chondrocytes, *L. sakei* LB-P12 may also inhibit NF-κB signaling pathway, thereby reducing HIF-2α-mediated MMP-13 expression. Collectively, *L. sakei* LB-P12 may improve joint health by attenuating inflammation and preventing cartilage degradation in osteoarthritis.

Under inflammatory conditions, chondrocytes increase the production of cartilage-degrading enzymes such as matrix metalloproteinases via upregulating several pathway including NF-κB and HIF-2α. In SW1353 chondrocytes, IL-1β significantly induced the mRNA levels of extracellular matrix degrading enzyme (*Mmp13*), and their upstream regulator HIF-2α compared to control (*p* < 0.05). As shown in Fig. 4E-4H, treatment of *L. sakei* LB-P12 resulted in significantly decrease of both *Mmp13* and *Epas1* (encoding HIF-2α), *Hif1a* expression was not significantly altered (data not shown). These findings suggest that *L. sakei* LB-P12 may alleviate the inflammation and cartilage degradation by regulating NF-κB/HIF-2α in macrophages and chondrocytes (Fig. 5).

## Discussion

Over the past few decades, OA has continued to increase worldwide, and has become a significant burden and major challenge for public health globally. Treatment of OA includes pharmacological, non-pharmacological, and surgical treatments, but all these treatments are conservative and cannot be cure the disease progression. Notably, long-term treatment of the medications may cause side effect including liver damage, stomach irritation, increasing blood pressure, and kidney disease. Probiotics represent a promising avenue for safe and natural alternatives to manage host health, and offer many advantages including anti-inflammatory, antimicrobial, anti-oxidant, and immunomodulatory properties. In the present study, we investigated for the first time on the positive effect of *L. sakei* LB-P12 on the treatment of OA in *in vitro* and *in vivo*.

To explore the potential effect of *L. sakei* LB-P12 on OA, MIA-induced rat model were used in this study. MIA injection is known to mimic human OA by inducing progressive disruption of cartilage, elevating plasma inflammatory cytokines, and causing joint pain [29-31]. Therefore, this model has been widely used for studying pharmacological effects on OA-related pathogenesis. In the present study, the treatment of *L. sakei* LB-P12 resulted in a dose-dependent pain relief in the MIA-induced OA model, as revealed by weight bearing analysis. Pain is the main symptom of OA, and it is the critical reason OA patients seek medical help [32]. Although the underlying mechanisms of OA pain are still not fully understood, pain is known to be closely associated with tissue damages, inflammations, or disorders of the nervous system [33, 34]. In histological and Micro-CT analysis data, we observed that treatment of *L. sakei* LB-P12 ameliorate the levels of articular cartilage degradation, and restore the bone mineral density (BMD) and bon volume (BV) compared to MIA group. These results indicate that administration of *L. sakei* LB-P12 ameliorate OA-associated pain with histological improvement.

Both systemic and local inflammation in synovial tissue were recently associated with progression of joint space narrowing and severity of pain in OA. In particular, IL-6, an proinflammatory cytokine, has been reported to be positively correlated with pain [35], while negatively correlated with joint function [36]. Plasma levels of the arachidonic acid metabolites prostaglandin E2 (PGE_2_) and 15-hydroxyeicosatetraenoic acid, both of which function as lipid mediators of inflammation, have been shown to be useful in distinguishing symptomatic osteoarthritis patients from healthy controls [37]. PGE_2_ can also exert many pathological effects that suppress chondrocyte proliferation and inhibit extracellular matrix (ECM) synthesis in the pathogenesis of OA [38]. Previous studies have shown that *L. sakei* exhibits anti-inflammatory properties in several inflammatory disease, including atopic dermatitis, colitis, and obesity [39-41]. Thus, we investigated whether *L. sakei* LB-P12 has anti-inflammatory effects on joint inflammation in the setting of OA. In this study, we found that administration of *L. sakei* LB-P12 decreased plasma levels of IL-6 and PGE_2_ as well as reducing *Il1b* expression in local synovial tissues in the MIA-induced rat model. These results indicated that *L. sakei* LB-P12 can improve OA-associated pain and cartilage degradation, at least in part with anti-inflammatory effects.

We also conducted *in vitro* experiments using two cell lines (Raw264.7 murine macrophage and SW1353 human chondrocytes), reflecting two critical cell types present in the joint tissue. qRT-PCR analysis data indicated that *L. sakei* LB-P12 inhibit the expression of proinflammatory cytokines including *Il6* in both macrophages and chondrocytes that induced by either LPS or IL-1β.

In OA, the NF-κB signaling pathway has extensively studied, and reported to have central role in intrinsic inflammatory responses in macrophages and chondrocytes. The NF-κB signaling pathway is likely activated by pathogen associated molecular patterns (PAMPs, *i.e.*, LPS) and damage associated molecular patterns (DAMPs, including products of extracellular matrix damage) through pattern-recognition receptor (PRR) including Toll-like receptor. Indeed, studies have shown that inflammatory pathways triggered by PRR signaling are activated in the joint both in OA patients and animal models [42, 43]. Here we found that *L. sakei* LB-P12 significantly inhibit the expression of NF-κB in both macrophages and chondrocytes.

A recent studies have demonstrated that the activation of NF-κB signaling pathways is also involved in the expression of matrix metalloproteinase (MMPs) through hypoxia-inducible factor 2α (HIF-2α) [44, 45]. MMPs is the enzyme that can degrade ECM, and the degradation of ECM within synovial joints is manifested by severe pain in patient with OA [46]. Among the various types of MMPs, MMP-13, primarily produced from chondrocytes, has been thought to be a key enzyme contribute to degenerative process during OA pathogenesis [46]. HIF-1α and HIF-2α has about 50% homologous, but there are functional differences between these in chondrocytes in the pathogenesis of OA. HIF-2α is a catabolic transcription factors that directly induces the expression of MMPs which degrade extracellular matrix (ECM), whereas HIF-1α acts as survival factor by enhancing ECM synthesis and inhibiting apoptosis in chondrocytes [28, 47]. In this study, treatment of *L. sakei* LB-P12 was resulted in significantly decrease of *Mmp13* expression in SW1353 human chondrocytes. Given that *L. sakei* LB-P12 inhibit the expression of NF-κB, and it is also critical transcriptional regulator of *Epas1* (encoding HIF-2α) in OA development, we hypothesized that *L. sakei* LB-P12 may regulate *Mmp13* expression through NF-κB/HIF-2α pathways in chondrocytes. The data showed that *L. sakei* LB-P12 treatment significantly down-regulated of HIF-2α expression in IL-1βstimulated chondrocytes. These results suggest that *L. sakei* LB-P12 may inhibit the inflammation and cartilage degradation by targeting NF-κB/HIF-2α signaling pathways in macrophages and chondrocytes.

Probiotics are functional food and are known to has beneficial effects through regulating gut microbiome, intestinal barrier function, and immune responses in many inflammatory diseases. In the present study, we demonstrated that *L. sakei* LB-P12 may inhibit the inflammatory and catabolic pathways by regulating NF-κB and HIF-2α. However, the mechanisms by which the crosstalk between *L. sakei* LB-P12 and host cells including macrophages and chondrocytes in the joints should be further studied in future studies.

Collectively, our findings suggest that *L. sakei* LB-P12 administration effectively attenuated MIA-induced OA by inhibiting cartilage degradation, inflammation and reducing pain. Mechanistically, *L. sakei* LB-P12 may improve osteoarthritis through regulation of the NF-κB/HIF-2α/HIF-1α signaling pathway in chondrocytes and macrophages (Fig. 5). Therefore, *L. sakei* LB-P12 has potential as a probiotic for mitigating OA progression.

## Supplemental Materials

Supplementary data for this paper are available on-line only at http://jmb.or.kr.
