# Supplementary material for: Latilactobacillus sakei LB-P12 Ameliorates Osteoarthritis by Reducing Cartilage Degradation and Inflammation via Regulation of NF-κB/HIF-2α Pathway
Source: J Microbiol Biotechnol. 2025 May 2;35:e2504013. doi: 10.4014/jmb.2504.04013 (PMC12089955; doi:10.4014/jmb.2504.04013)
Supplement: Supplementary file 1 [file jmb-35-e2504013-supple.pdf]

J. Microbiol. Biotechnol. 2025. 35: e2504013
https://doi.org/10.4014/jmb.2504.04013

Supplementary Figure and Table

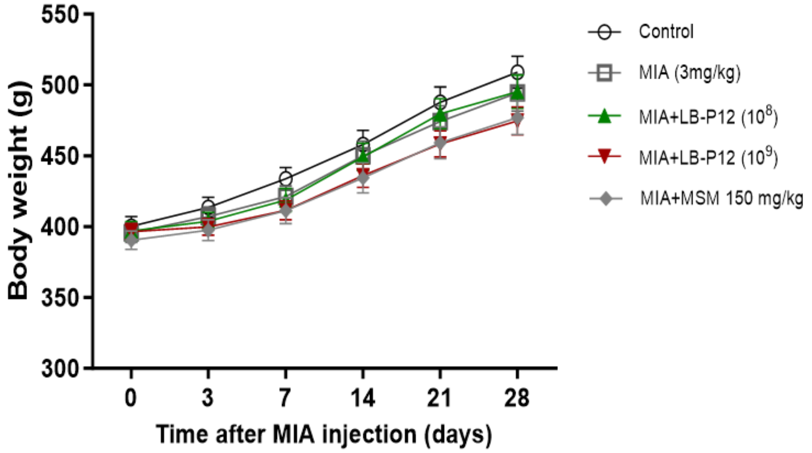

Supplementary Fig. S1. Body weights throughout the experimental period. *L. sakei* LB-P12 did not affect body weight in the OA rat model. Although the body weights of all rats increased during the experiment, the weekly measurements did not exhibit any significant variations between the study groups.

Table S1. Forward and reverse sequences of q-PCR primers in this study.

Gene	Primer Direction	Primer Sequence	Reference
In vitro			
mNos2	Forward	GAGACAGGGAAGTCTGAAGCAC	[1]
	Reverse	CCAGCAGTAGTTGCTCCTCTTC	
mIl1b	Forward	AAGGAGAACCAAGCAACGACAAA	[2]
	Reverse	CAGAGCCACAATTCCCTTTCTA	
mTnf	Forward	ATGGCCTCCCTCTCATCAGT	[3, 4]
	Reverse	CGCACTAGGTTTGCCGAGTA A	
mIl6	Forward	CCCCAATTTCCAATGCTCTCC	
	Reverse	CGCACTAGGTTTGCCGAGTA	
mNfkb1	Forward	ATTTGAAACACTGGAAGCACGG	[5]
	Reverse	CCGCCTTCTGCTTGTAGATAGG	
mGapdh	Forward	GTGTTCCTACCCCCAATGTGT	[6]
	Reverse	ATTGTCATACCAGGAAATGAGCTT	
hRELA	Forward	TCCGTTATGTATGTGAAGGC	[7]
	Reverse	TTTGCTGGTCCCACATAGTTGC	
hIL6	Forward	AGACAGCCACTCACCTCTTCAG	[8]
	Reverse	TTCTGCCAGTGCCTCTTTGCTG	
hHIF2α	Forward	TGCAGACCTTGTCTTGAAGGTG	
	Reverse	TGCAGACCTTGTCTTGAAGGTG	
hMMP13	Forward	GCGTCATGCCAGCAAATTC	[9]
	Reverse	TCCCCTACCCCGCACTTC	
hHIF1α	Forward	ACCATGCCCCAGATTCAGGA	[10]
	Reverse	ATCAGTGGTGGCAGTGGTAGTGGT	
hGAPDH	Forward	GTCGGAGTCAACGGA TTTGG	[11]
	Reverse	GGGTGGAATCAATTGGAACA	
in vivo			
Il1b	Forward	TGACCCATGTGAGCTGAAAG	[12]
	Reverse	GGGATTTTGTCGTTGCTTGT	
Mmp13	Forward	ACCATCCTGTGACTCTTGCG	[13]
	Reverse	TTCACCCACATCAGGCACTC	
Gapdh	Forward	ACTCCCATTCTTCCACCTTTG	[14]
	Reverse	CCCTGTTGCTGTAGCCATATT	

References

1. Steiger S, Kumar SV, Honarpisheh M, Lorenz G, Günthner R, Romoli S, *et al.* 2017. Immunomodulatory Molecule IRAK-M Balances Macrophage Polarization and Determines Macrophage Responses during Renal Fibrosis. *The Journal of Immunology.* **199:** 1440-1452.
2. Luo Q, Liu M, Tan Y, Chen J, Zhang W, Zhong S, *et al.* 2022. Blockade of prostaglandin E2 receptor 4 ameliorates peritoneal dialysis-associated peritoneal fibrosis. *Frontiers in Pharmacology.* **13**.
3. Seon Y-G, Jeong JM, Yoon J-S, Noh J, Im SK, Bang S-P, *et al.* 2023. A Study of the Antioxidant and Anti-Inflammatory Effects of Dusokohwaeum. *J Acupunct Res.* **40:** 356-367.
4. Luo J, Tian Z, Song F, Ren C, Liu W. 2024. Dual-specificity phosphatase 5-mediated fatty acid oxidation promotes Mycobacterium bovis BCG -induced inflammatory responses. *Experimental Cell Research.* **434:** 113869.
5. Hao J, Shi FD, Abdelwahab M, Shi SX, Simard A, Whiteaker P, *et al.* 2013. Nicotinic receptor β2 determines NK cell-dependent metastasis in a murine model of metastatic lung cancer. *PLoS One.* **8:** e57495.
6. Yoshitomi H, Kobayashi S, Miyagawa-Hayashino A, Okahata A, Doi K, Nishitani K, *et al.* 2018. Human Sox4 facilitates the development of CXCL13-producing helper T cells in inflammatory environments. *Nature Communications.* **9:** 3762.
7. Kim BR, Yoon K, Byun HJ, Seo SH, Lee SH, Rho SB. 2014. The anti-tumor activator sMEK1 and paclitaxel additively decrease expression of HIF-1α and VEGF via mTORC1-S6K/4E-BP-dependent signaling pathways. *Oncotarget.* **5:** 6540-6551.

8. Zhang H, Lv X, Kong Q, Tan Y. 2022. IL-6/IFN-γ double knockdown CAR-T cells reduce the release of multiple cytokines from PBMCs in vitro. *Hum Vaccin Immunother.* **18:** 1-14.

9. Zhou N, Lin X, Dong W, Huang W, Jiang W, Lin L, *et al.* 2016. SIRT1 alleviates senescence of degenerative human intervertebral disc cartilage endo-plate cells via the p53/p21 pathway. *Sci Rep.* **6:** 22628.

10. Coimbra IB, Jimenez SA, Hawkins DF, Piera-Velazquez S, Stokes DG. 2004. Hypoxia inducible factor-1 alpha expression in human normal and osteoarthritic chondrocytes11Supported by NIH/NIAMS Program Project grant (AR-39740) to S.A.J. I. C. was supported by a fellowship from Fundacao de Amparo‘a Ciencia do Estado de Sao Paulo. *Osteoarthritis and Cartilage.* **12:** 336-345.

11. Song M, Shim J, Song K. 2024. Oral Administration of Lactilactobacillus curvatus LB-P9 Promotes Hair Regeneration in Mice. *Food Sci Anim Resour.* **44:** 204-215.

12. Shim CY, Song B-W, Cha M-J, Hwang K-C, Park S, Hong G-R, *et al.* 2014. Combination of a peroxisome proliferator-activated receptor-gamma agonist and an angiotensin II receptor blocker attenuates myocardial fibrosis and dysfunction in type 2 diabetic rats. *Journal of Diabetes Investigation.* **5:** 362-371.

13. Gu R, Huang Z, Liu H, Qing Q, Zhuan Z, Yang L, *et al.* 2019. Moracin attenuates LPS-induced inflammation in nucleus pulposus cells via Nrf2/HO-1 and NF-κB/TGF-β pathway. *Biosci Rep.* **39**.

14. Li Y-S, Yang R-R, Li X-Y, Liu W-W, Zhao Y-M, Zu M-M, *et al.* 2024. Fluoride impairs vascular smooth muscle A7R5 cell lines via disrupting amino acids metabolism. *Journal of Translational Medicine.* **22:** 528.
